# Evaluation of rotator cuff muscle strength in healthy individuals

**DOI:** 10.1590/1413-78522015230300350

**Published:** 2015

**Authors:** Paulo José Oliveira Cortez, José Elias Tomazini

**Affiliations:** 1 Faculdade de Medicina de Itajubá (FMIt), Itajubá, MG, Brasil. Universidade Estadual Paulista Júlio de Mesquita Filho (FEG/UNESP), Departamento de Mecânica, Guaratinguetá, SP, Brazil

**Keywords:** Rotator cuff, Shoulder joint, Muscle strength

## Abstract

**OBJECTIVE:**

To compare the strength generated by the rotator muscles of the shoulder joint between the right upper limb and left upper limb among healthy individuals.

**METHODS:**

To evaluate the muscle strength of upper limbs from isometric contractions in the horizontal direction (rotation) an isometric dynamometer was used, equipped with transducers, signal conditioning, a data acquisition board, and finally, a computer. Study participants were 22 male military subjects, aged between 18 and 19 years old, body mass between 57.7 and 93.0 kg (71.8 ± 9.45 kg) and height between 1.67 and 1.90 m (1.75 ± 0.06 m), healthy and without clinical diseases or any type of orthopedic injury in the muscle skeletal system.

**RESULTS:**

The internal rotation in the right upper limb (RUL) was higher than the average strength of internal rotation in the left upper limb (LUL) (p = 0.723). The external rotation strength in RUL was lower than the average strength of external rotation in the LUL (p=0.788). No statistical difference was observed by comparing the strength values of all isometric strength tests.

**CONCLUSION:**

For the sample and methodology used to assess muscle strength, there was no statistical difference between the strength generated by the muscles of the rotator cuff of the right and left upper limbs. *Experimental Study.*

## INTRODUCTION

The joint action of the shoulder rotator muscles promotes shoulder joint dislocations and act as important articular stabilizers.^1^ The rupture of the muscle-tendon units of the rotator cuff determines conditions of pain, instability or a combination of both simptons.^2^^,^ 3

Thus, these muscles are extremely important for the entire articular complex and needs further clinical care regarding its function and dysfunction. It has been observed that the decreased ability to exert efforts by the rotator muscles can create a variety of conditions, from inability to seemingly simple activities such as combing hair and brushing teeth until the complete loss of funcionality.^4^^,^ 5

The precise knowledge of muscle strength level of an individual is important for both for the evaluation of occupational functional capacity as well as an appropriate prescription for athletic and rehabilitation exercises.^6^

Rabin and Post^7^ compared the evaluation by manual methods and isokinetic instrumental evaluation of the bending momentum and external rotator of the shoulder before and after surgery.

It was found that with the manual method, the estimated momentum was higher, however, this increase was not evident with the instrumental evaluation.

According to Hsu et al.^8^ by understanding that the gain of muscular strength improve the functionality of the patient, it is important that muscle strength measurement methods are accurate and reliable. Hence the need for equipment that can assist the accurate assessment of muscle strength and experimental analysis of engineering be possible. This analysis refers to applications where the measurement provided by an instrument is used for a post-measurement analysis for determination of a parameter, model, and / or its validation.^9^

According to Weinstein and Buckwalter^10^ measurement devices extend the possibilities of the physical examination, particularly in the case of complex musculoskeletal problems and during rehabilitation of patients with muscular asthenia or restriction of range of motion.

In a review study by Jaric^11^ on muscular strength tests it is noticed that most studies involving evaluation of strength or torque were performed in the lower limbs, suggesting new studies involving upper limbs. According to Dvir^12^ there is shortage of information on the functional connections of the shoulder girdle and factors involved in muscle strength in this region.

Comparative studies involving muscle strength as well as considerations about the dominance and non-dominance ratio have been studied by many researchers.^13^^-^ 16 However, little is known about this relationship when it comes to the shoulder rotators muscle groups.

Thus, the aim of this study was to compare the strength generated by the rotator muscles of the shoulder joint between the right and left upper limbs among healthy subjects. 

## METHODS

**Figure 1.**
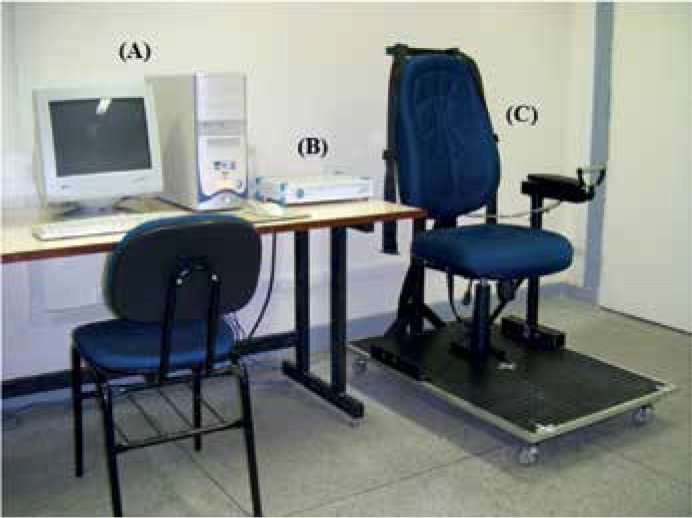
Acquisition system: (A) computer, (B) acquisition and signal conditioning model SPIDER 8 and (C) strength measurement station.

After approval by the Research Ethics Committee of *Fundação de Ensino e Pesquisa de Itajubá - Centro Universitário de Itajubá* and signing of the Free and Informed Consent, participated in the study 22 (twenty two) healthy male subjects, aged between 18 and 19 years old, body mass between 57.7 and 93 kg (71.8 ± 9.45 kg) and height between 1.67 and 1.90 m (1.75 ± 0.06 m), with no history orthopedic disease or any kind of injury to the musculoskeletal system. All study subjects were soldiers of the Air Force Infantry Battalion (BINFA), physically active and performing regular military activity at Escola de Especialistas da Aeronáutica (EEAR), Guaratinguetá, SP, Brazil. Strength tests were performed at the Laboratório de Resistência dos Materiais, Departamento de Mecânica da UNESP, Guaratinguetá unit, SP, Brazil, from device designed and built by Cortez.^17^ (Figure 1)

According to the recommendations of The American Society of Exercise Physiologists (ASEP),^6^ standardized instructions to individuals were given aiming to reduce the margin of error during testing. Three maximal voluntary contractions were requested (MVC) for 10 seconds, with an interval of 30 seconds between each MVC. The position of the upper limb for this test complied with the Bohannon^18^ guidelines where the shoulder joint remained in the neutral position, the elbow flexed approximately at 90° and the forearm in neutral position. Subjects were instructed to perform a maximal voluntary contraction aiming to perform medial and lateral rotation movements of the shoulder joint (horizontal effort). After application of the Anderson-Darling normality test, data were analyzed. In order to compare the means of variables analyzed, the concept of quartile and the Student *t*-test with statistical significance α=5% (p<0.05) were initially used.

Figure 1 illustrates the system formed by the device built, the acquisition and signal conditioning system, and a computer. 

## RESULTS

Table 1 shows the data obtained in strength tests in the right arm and the left upper limb.

**Table 1.** Results obtained from the tests: Internal rotation of the left upper limb (IR LUL) and internal rotation of the right upper limb (IR RUL), external rotation of the left upper limb (ER LUL) and external rotation of the right upper limb (ER RUL).

Variable	Mean	Median	Minimum	Maximum	Q1	Q3
IR RUL	164.84	160.02	127.74	236.16	144.07	179.00
IR LUL	161.88	158.85	124.56	239.12	138.85	175.57
ER RUL	102.09	101.54	76.53	130.51	90.30	114.92
ER LUL	103.36	102.30	80.02	135.33	90.27	116.78

Q1: first quartile; Q3: third quartile.

**Figure 2.**
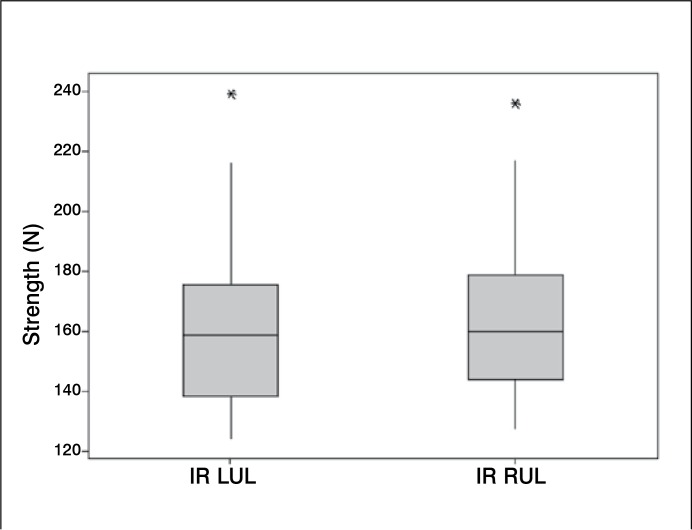
Boxplot showing data obtained at the tests: Internal rotation of left upper limb (IR LUL) and right upper limb (IR RUL).

Figure 2 illustrates the boxplots obtained with the data of the variables: Internal rotation of the left upper limb (IR LUL) and internal rotation of the right upper limb (IR RUL). It was observed that the values (mean, median, first quartile and third quartile) obtained for IR RUL are higher than the values of IR LUL.

**Table 2.** Results obtained from variables comparing the following tests: strength (F), internal rotation (IR) and external rotation (ER) between right upper limb (RUL) and left upper limb (LUL).

Variable	Mean	St. Dev	Minimum	Maximum	*p*-Value
IR RUL	164.84	27.02	127.74	236.16	0. 723
IR LUL	161.88	28.03	124.56	239.12	
ER RUL	102.09	14.61	76.53	130.51	0. 788
ER LUL	103.36	16.36	80.02	135.33	

Statistical significance = p< 0.05; St. Dev = Standard deviation.

**Figure 3.**
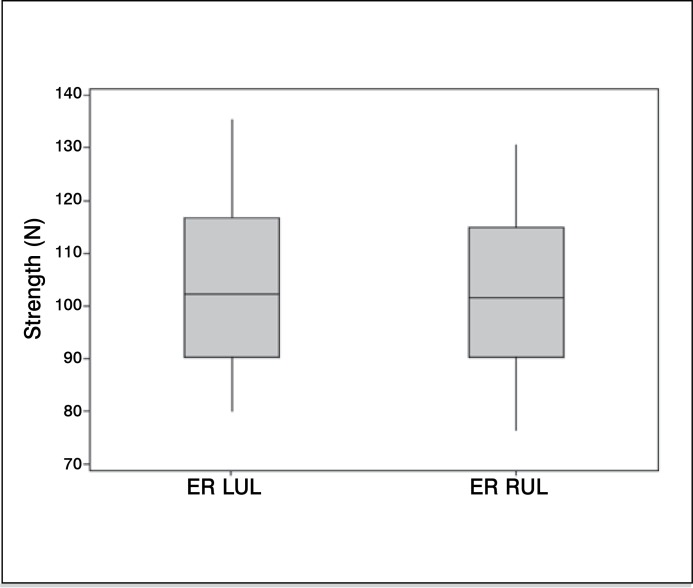
Boxplot showing data obtained at the tests: External rotation of left upper limb (ER LUL) and right upper limb (IR RUL).

Figure 3 illustrates the boxplots obtained with the data of the variables: External rotation of the left upper limb (ER LUL) and external rotation of the right upper limb (ER RUL). It is observed that the values (mean, median, first quartile and third quartile) obtained for ER RUL are higher than the values of ER LUL.

Once the normality of the data was checked by the Anderson-Darling test, the Student t-test was used to compare the muscle strength between RUL and LUL with the values obtained in the three tests applied. The Student t-test is widely used by researchers such as Simões et al.,^19^ Fleming and McGregor,^16^ and Bohannon^20^ in the investigation of differences in the generation of strength between the dominant and non-dominant limbs.

The mean strength values (N), standard deviations, minimum values (N) and maximum values (N) of the variables: mean maximum strength of IR RUL, mean maximum strength of IR LUL, mean maximum strength of ER RUL, average maximum strength of the ER LUL and the values obtained (p-value) for comparisons of mean strength between strength tests for RUL and LUL through the Student t-test (assuming p <0.05). Table 2 presents the data obtained from comparing the values.

## DISCUSSION

The mean internal or medial rotation strength in the RUL was higher than the mean internal or medial rotation strength in LUL. However, by comparing the means, a statistical similarity between values was noticed (p=0.723). As for the external or lateral rotation test, the mean lateral rotation strength in the RUL was lower than the average lateral rotation strength in LUL. However, when comparing the figures, it was found that there was no statistical difference between the mean strengths in this test.

With the observation of p-value, it can be concluded that there were no statistical differences in the strength values ​​of all tests performed in this study. The p-value was always higher than 0.05.

Coincidentally, all subjects in the present study reported right dominance, i.e., were right-handed and used preferably the right upper limb (RUL) in daily activities. It is important to point out that this dominance and non-dominance relationship is not yet well understood.

The study population, for convenience, has been chosen in an attempt to homogenize the sample regarding demographics, age, frequency and intensity of physical activity, diet and rest periods.

The data of this study agrees with the results found by Shklar and Dvir^21^ where the muscle strength of the dominant limb in the shoulder joint rotation tests was higher than in the non-dominant side. However, statistically, there was no difference between them.

In his study, MacKinnon^14^ built a device for isometric muscle strength analysis of the upper right limb in different positions, using a load cell. The study included eight subjects (five men and three women) aged 20-43 years. Information of participating subjects such as occupational activity and physical activity as well as general health of the subjects, however, were not informed. The average strength values ​​(N) found by this author vary according to the position of the subject during testing. For example, in the sitting position, the averages for efforts in the sagittal plane (flexion) ranged between 75 and 204 N. When the subjects were tested in a standing position, the averages ranged from 99 to 241 N. In this study, there was no statistical difference between the strengths produced by muscle contraction of the dominant limb as compared with the strengths produced by the non-dominant limb.

In the study by Fleming and McGregor^16^ it was found that muscle strength of the dominant limb was significantly higher when compared to the non-dominant limb. These authors confirmed the statistical difference between the dominant and non-dominant limbs in professional tennis players.

Ertem et al.^15^ investigated the dominance relationship, body mass and age as determining factors in the functional evaluation process of hands. Regarding the assessment of strength between the dominant and non-dominant limbs, the authors found that the dominant side generated greater strength than the non-dominant limb.

Respecting the studies presented above, it is clear that there is no consensus among researchers about the dominance relationship and that further studies are essential to better understand the phenomena involved in the generation of muscle strength.

## CONCLUSION

We conclude that for the studied sample and methodology used to assess muscle strength, there was no statistical difference in the comparison of the strength generated by the rotator muscles of the upper shoulder of the right and left upper limbs. Moreover, the strength measuring device called strength measurement station was efficient for the measurement of muscle strength.
